# Supplementary material for: Exact Primary Tumor Location in mCRC: Prognostic Value and Predictive Impact on Anti-EGFR mAb Efficacy
Source: Cancers (Basel). 2022 Jan 21;14(3):526. doi: 10.3390/cancers14030526 (PMC8833757; doi:10.3390/cancers14030526)
Supplement: Supplementary file 1 [file cancers-14-00526-s001.zip › cancers-1520354-SI.pdf]

Figure S1. Consort diagram.

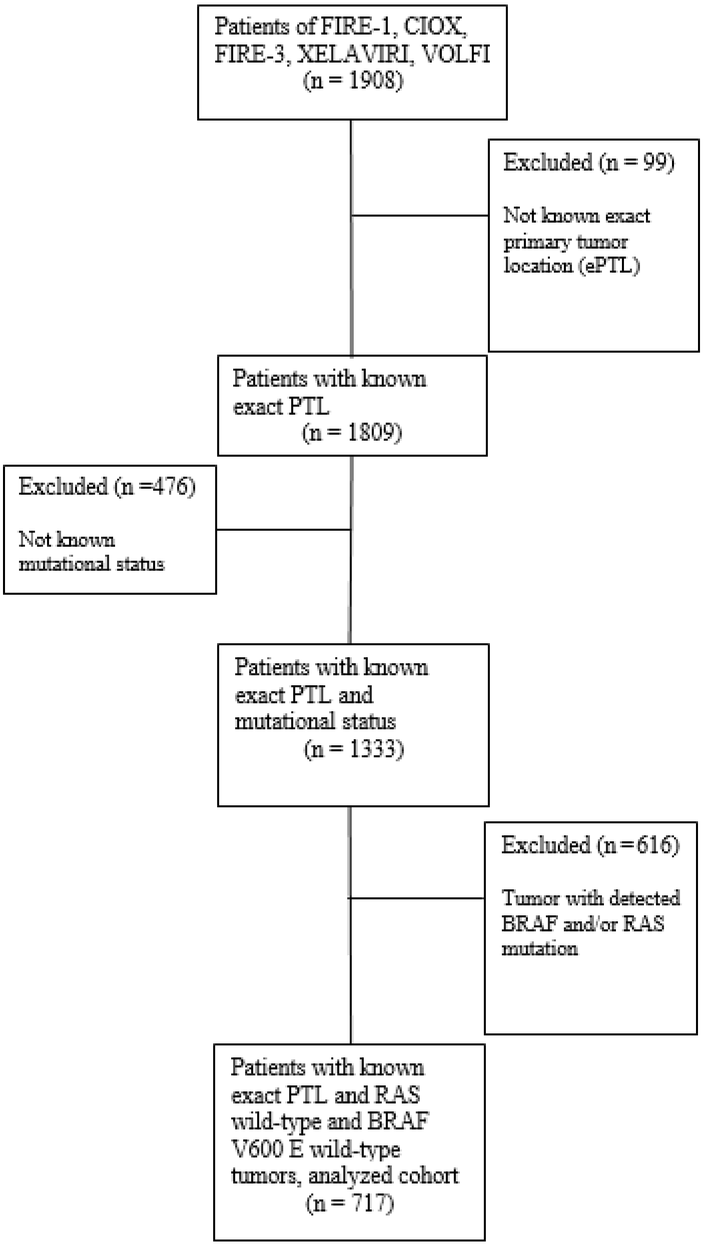

Legend: PTL=primary tumor location.

Figure S2. Diagram of the study population.

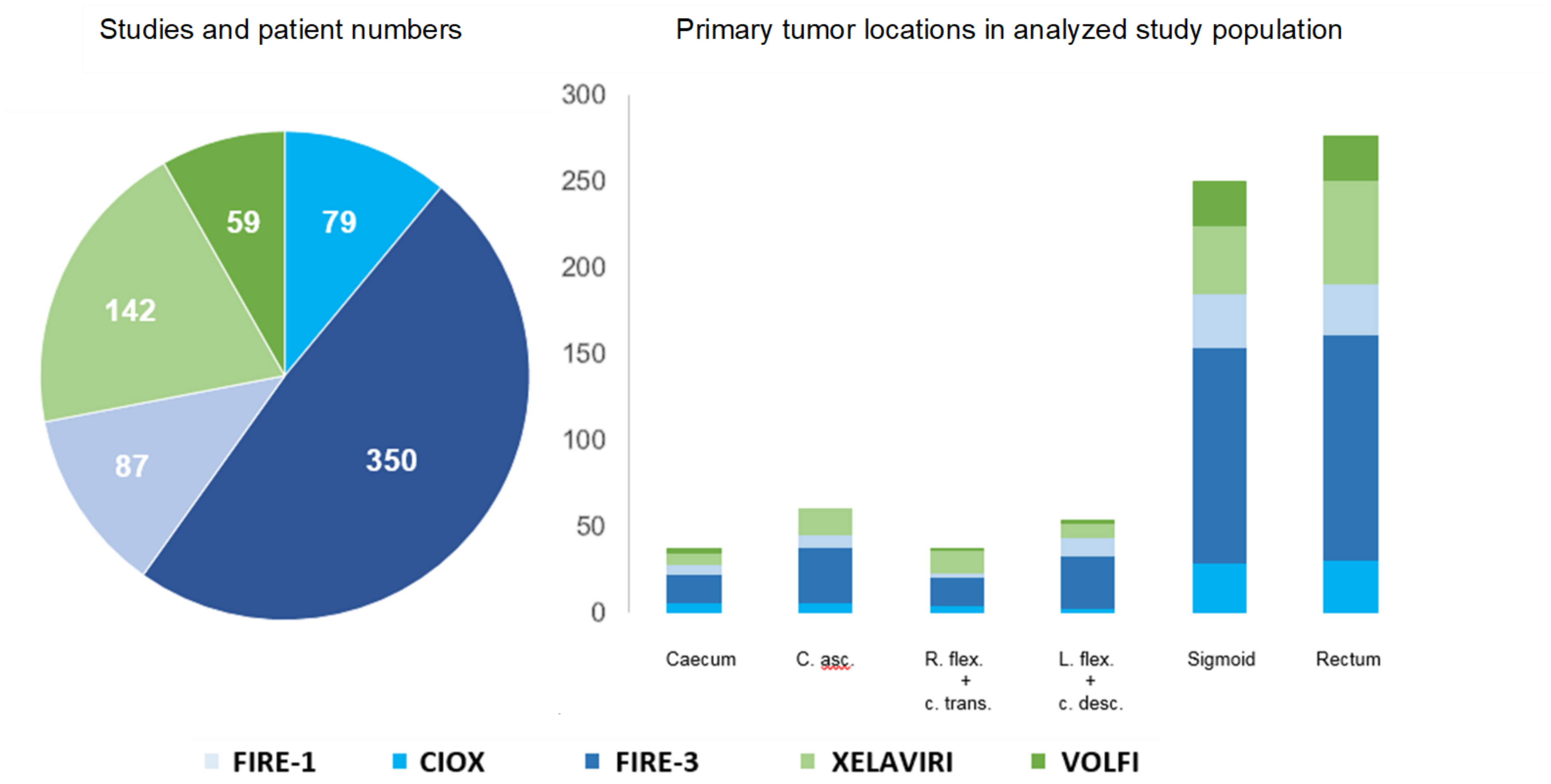

Legend: Frequency of primary tumor location in the analyzed population. C. asc.= ascending colon; c. desc.=descending colon; c.trans=transverse colon; l. flex=left flexure; r. flex.=right flexure.

Figure S3. Baseline and tumor characteristics.

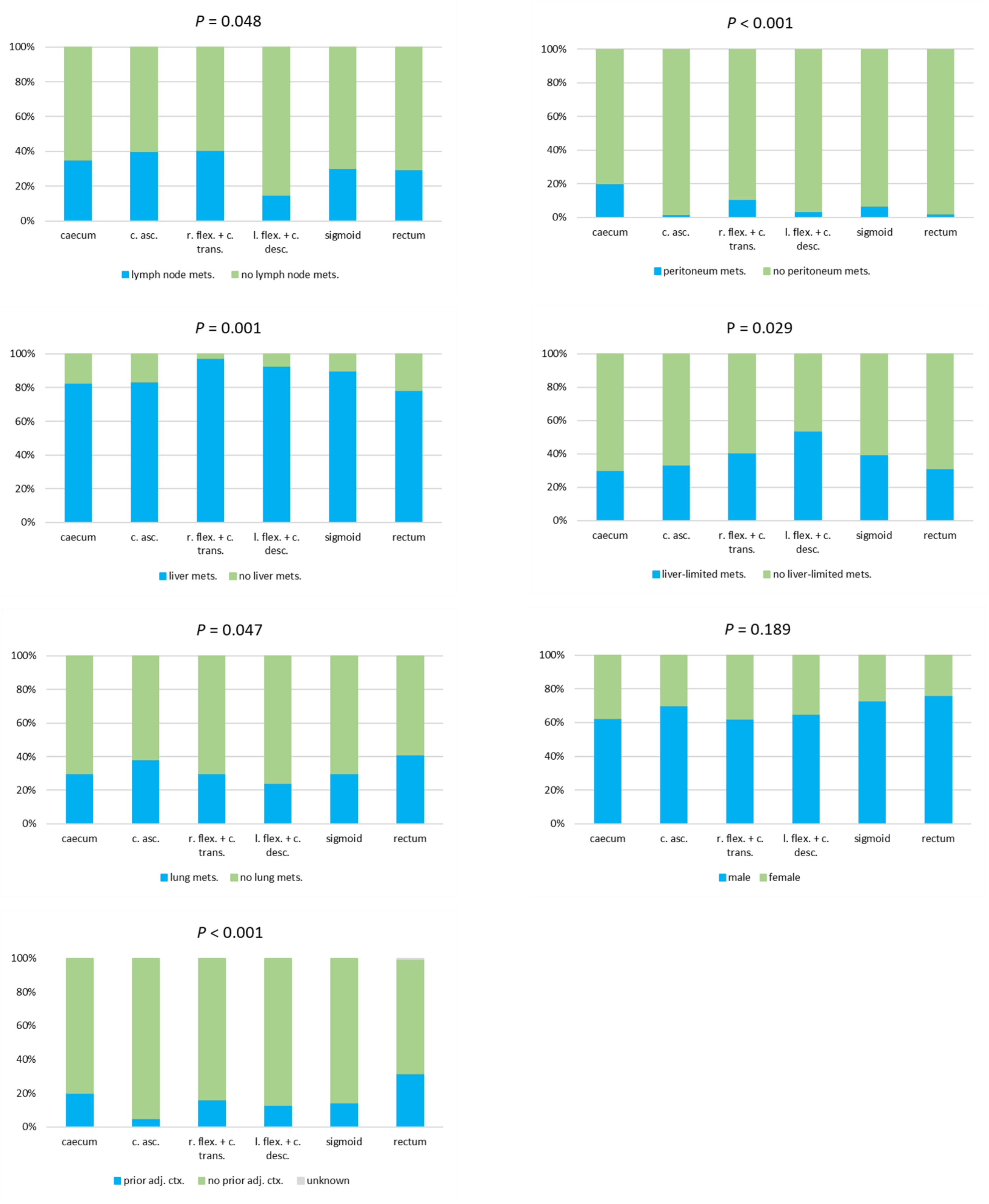

Legend: adj.=adjuvant; c. asc.=ascending colon; c. desc.=descending colon; c. trans.=transverse colon; ctx.=chemotherapy; l. flex.=left flexure; r. flex.=right flexure; mets.=metastases.

Figure S4. Objective response rate according to primary tumor location and received treatment.

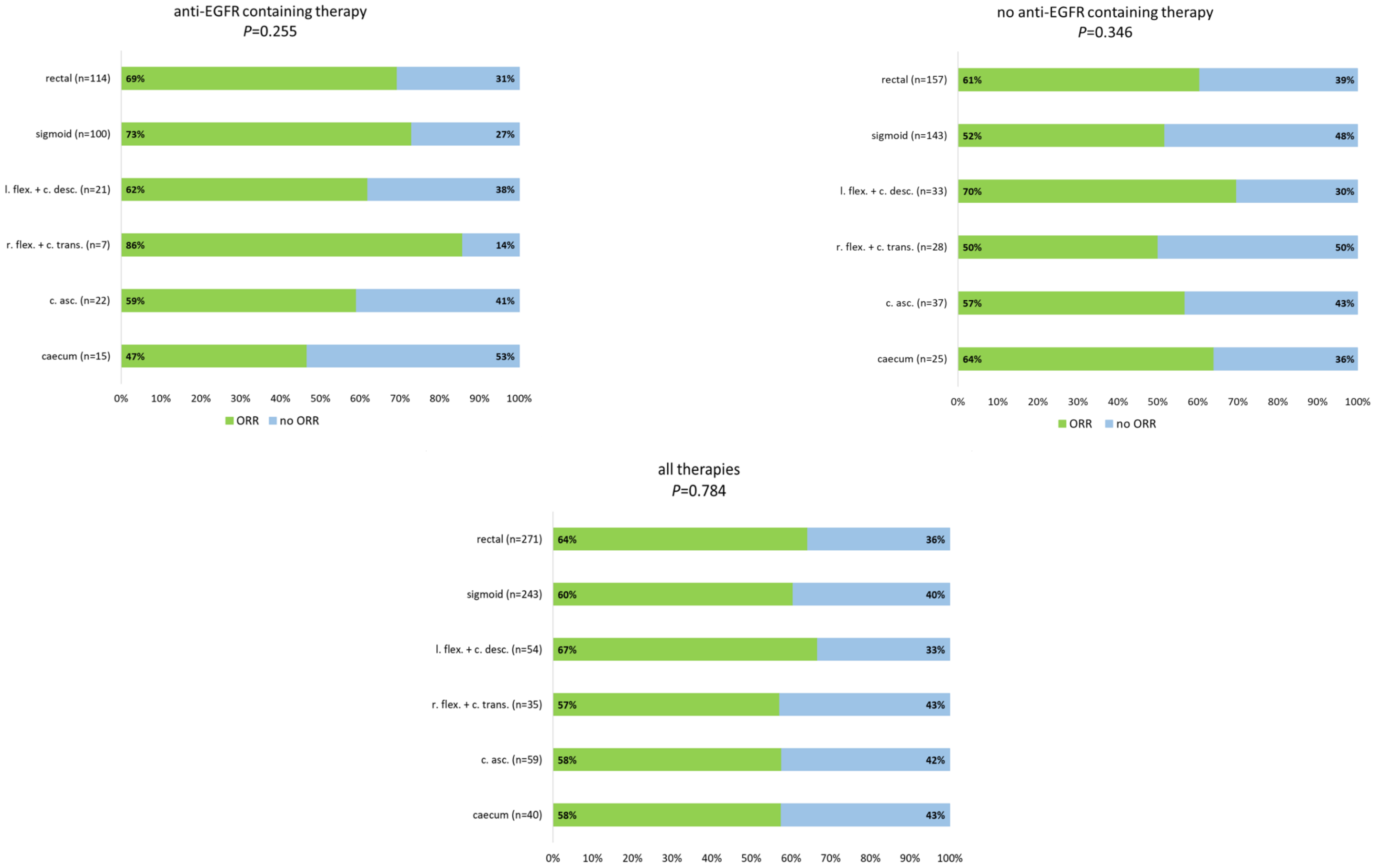

Legend: c. asc.=ascending colon; c. desc.=descending colon; c. trans.=transverse colon; l. flex.=left flexure; ORR=objective response rate; r. flex.=right flexure.
